# Illinois State Dental Society

**Published:** 1874-06

**Authors:** 


### ILLINOIS STATE DENTAL SOCIETY.

The tenth annual meeting of the Illinois State Dental Society was held at Jacksonville, on the 12th and 15th inclusive, of May.

Dr. C. Stoddard Smith, of Springfield, President; and Dr. E. R. E. Koch, Recording Secretary.

There were about seventy members present, and in addition to these quite a number of visitors, some of them dentists; but many of them from outside of the profession, the latter manifested great interest in the proceedings. We have never had so many of this class of persons in a dental meeting before. Such a fact portends great good; it not only shows that there is a warm interest in that community in behalf of the dental profession, but such attention will serve as a stimulus upon the profession wherever it is manifested.

The subjects presented and discussed were given in the last number of the REGISTER. They elicited several valuable papers and much interesting discussion, a report of which we will not attempt to give here, as the whole proceedings will be published in the volume of transactions. It was our privilege to be present at the meeting, and we must say that we have not seen a more efficient, harmonious and persevering body anywhere in the profession. Each one seemed determined that the greatest good should result from their efforts. Clinics were held during the forenoon of each day, for which the arrangements were very thorough, thus giving facilities for the realization of very gratifying results. Could clinics always be as well and satisfactory conducted as here, there would certainly be less criticism as to the practicability of holding them at all dental meetings. This society embraces a large share of the best men of the profession in Illinois, and with the spirit they now possess we can scarcely conceive of any great results for the good of the profession they may not accomplish Much would be gained in the way of knowledge, sympathy and stimulus, by having intelligent and cultivated people attend dental societies upon all occasions so far as practicable.

